# Fully automated macromolecule suppressed single voxel glutamate spectroscopy (FAMOUS SVGS)

**DOI:** 10.1186/s12967-016-0970-1

**Published:** 2016-07-26

**Authors:** Ravi Prakash Reddy Nanga, Hari Hariharan, Ravinder Reddy

**Affiliations:** Department of Radiology, Center for Magnetic Resonance and Optical Imaging, University of Pennsylvania, B1 Stellar-Chance Laboratories, 422 Curie Boulevard, Philadelphia, PA 19104-6100 USA

**Keywords:** Single voxel ^1^H MRS, Glutamate quantification

## Abstract

**Purpose:**

The aim of the study was to develop and validate a new localized ^1^H MRS pulse sequence and automated post-processing software for the quantification of brain Glutamate (Glu) in clinical conditions at 7.0T in order to get reliable and reproducible results for acute intervention studies.

**Methods:**

Here we describe a new localized proton MRS method “Fully Automated MacrOmolecUle Suppressed Single Voxel Glutamate Spectroscopy (FAMOUS SVGS)” for measuring Glu. FAMOUS SVGS method consists of a new pulse sequence with optimized switchable water, metabolites and outer volume suppression modules, as well as a frequency selective inversion pulse and automated post-processing of the five spectra obtained. FAMOUS SVGS method was first validated with glutamate phantoms and then validated with test–retest repeatability studies in the occipital cortex of five normal volunteers at 7.0T.

**Results:**

Glutamate concentrations estimated from phantoms with FAMOUS SVGS method correlated well with actual concentrations. Test–retest repeatability studies in human brain in vivo yielded less than 0.3 mM intra-subject variations in Glu concentrations.

**Conclusions:**

FAMOUS SVGS method enables Glu quantification in vivo at 7.0T with test–retest variability of less than 0.3 mM. We expect that we can reliably measure ≥0.5 mM change in glutamate due to any acute intervention.

## Background

Glutamate (Glu) is a major excitatory neurotransmitter that is present in abundance in the synaptic vesicles of the human brain [1]. It plays an important role in the day-to-day functions such as cognitive, learning and memory, a key metabolite in cellular metabolism, and helps in removal of ammonia from the body. Its quantification has gained significant importance due to its implications in many of the neuropsychological as well as neurodegenerative disorders. In these brain disorders, as the changes in the Glu levels are expected to be relatively small, it is important to have a non-invasive method that measures these small changes in Glu concentration with high degree of accuracy and repeatability.

Single voxel proton magnetic resonance spectroscopy (^1^H MRS) which is a safe, non-invasive method for the detection of human brain metabolites in vivo, has been widely applied for the study of Glu levels in many of the neurological disorders as well as in cases of insomnia and addiction disorders [2–14]. With the availability of 7.0T ultra-high field magnets, and the phased-array head coils, there is an improved resolution in the ^1^H MRS and appreciable gain in signal-to-noise ratio (SNR) over the lower field strength magnets [15]. In a previous acute gabapentin drug study done at 7.0T, the effect of drug produced detectable change in GABA but not for glutamate [16]. Based on literature reports and from our own experience, we feel the following issues might contribute to the glutamate quantification errors: (i) Eddy currents, (ii) Phased-array combination from the multi-channel receive head coils, (iii) Chemical shift artifacts, (iv) Phase correction, (v) Baseline correction, and (vi) Macromolecular contaminations. There are few test–retest studies done at 7.0T, where mean inter-subject variations in glutamate estimation are reported to be around 5 % [17, 18], but intra-subject test–retest variation for each individual in glutamate estimation is not reported. Our interest is in detecting the absolute quantification of glutamate for pre- and post acute interventions of an individual with an aim to detect ≥0.5 mM change in glutamate concentrations in clinical conditions such as temporal lobe epilepsy, cortical dysplasia, Alzheimer’s, Psychiatric disorders, etc.

This has motivated us to develop a new strategy to design a new pulse sequence as well as develop an automated processing pipeline with no user intervention to mitigate the above-mentioned issues with the single voxel ^1^H MR spectra, for improved repeatability of the Glu measurement.

In this study, we demonstrate a new method for quantification of the Glu at 2.35 ppm from the Fully Automated MacrOmolecUle Suppressed Single Voxel Glutamate Spectroscopy (FAMOUS SVGS). FAMOUS SVGS ^1^H MRS with an automated post-processing provides a simple spectrum with a flat baseline. FAMOUS SVGS method was validated in vitro using glutamate monosodium phantoms. We investigated in vivo efficacy of this method by acquiring the data from the voxel in occipital cortex from multiple subjects, at two-time points.

## Methods

### FAMOUS SVGS sequence

**Fig. 1:**
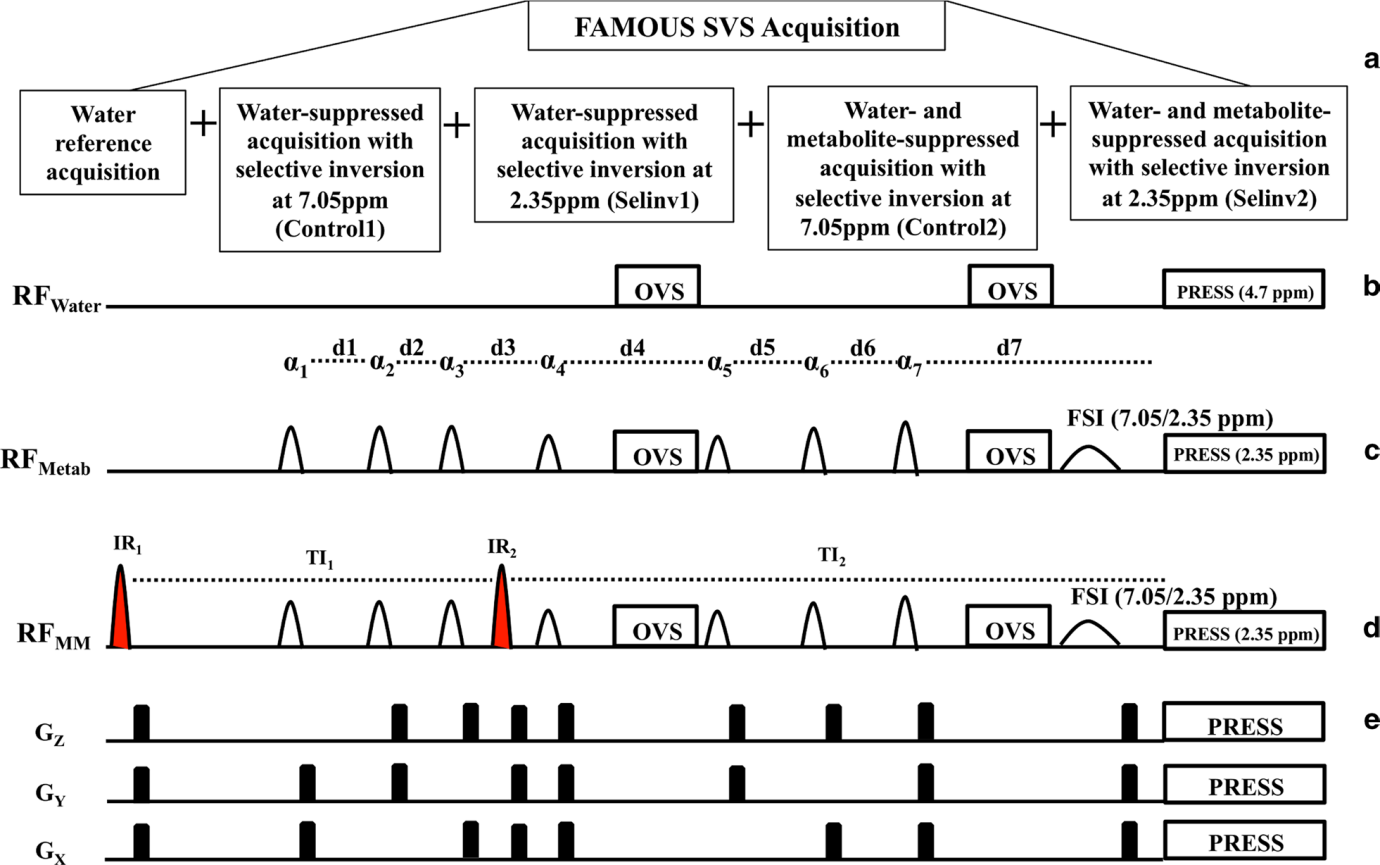
**a** FAMOUS SVS pulse sequence diagram with three acquisition blocks. **b** RF diagram for water reference acquisitions with all the saturation pulses off and gradients on along with optional OVS pulses. **c**, **d** RF diagram for water-suppressed control and inversion acquisition showing the seven water saturation pulses (α1–α7) along with the delays (d1–d7), two FSI pulse and optional OVS pulses. For the water-suppressed metabolite nulled control and inversion acquisition there are two additional inversion pulses (*IR*), one set at the beginning of the acquisition while the second one is set in between the third and fourth water saturation pulse **e** Gradients along x, y and z axes are all same for the three acquisitions

FAMOUS SVGS pulse sequence is shown in Fig. 1. The sequence consists of a water reference acquisition, two control acquisitions with selective inversion set at 7.05 ppm [“water suppressed” (Control1) and “water and metabolite suppressed” (Control2)], and two selective inversion acquisitions set at 2.35 ppm [“water suppressed” (Selinv1) and “water and metabolite suppressed” (Selinv2)]. The number of averages for the water reference acquisition and water-suppressed acquisitions can be independently set. The basic sequence has a magnetization preparation and single voxel acquisition blocks. The preparation block consists of double inversion for metabolite suppression, water suppression, outer volume suppression (OVS) and frequency selective inversion (FSI). The acquisition block consists of the standard PRESS sequence. The double inversion segment is designed with broad band adiabatic inversion pulses that do not excite water (pulse width = 18 ms, bandwidth = 3000 Hz (10 ppm centered at −1 ppm). The inversion delays are optimized to provide good inversion for metabolite T_1_ values between 1 and 2 s. Water suppression is performed using seven water suppression pulses (α1–α7); Gaussian shape with user-defined bandwidth and seven inter-pulse delays (d1–d7). Delay d4 is designed to be long enough (>20 ms) to accommodate up to 4 OVS pulses (3.5 ms radio frequency (RF) pulse (bandwidth = 5600 Hz) and 1.3 ms gradient spoiler). Delay d7 is designed to be long enough (>45 ms) to accommodate both the OVS and FSI pulses. The FSI pulse is a Gaussian pulse with its width calculated to provide 50 % inversion over a user defined inversion bandwidth (typically 60 Hz to cover the distance between the center peak of the triplet to the either side (7 Hz + 7 Hz) and an additional 45 Hz for shim in-homogeneities). The water reference acquisition is performed with all spoiler gradients and OVS RF pulses without any water suppression, double inversion or FSI pulses. This is essential since we use the water reference spectrum to correct eddy current induced dynamic phase shifts for all the five spectra. “Water suppressed” spectra are acquired with water suppression, OVS and FSI pulses. “Water and metabolite suppressed” spectra are obtained with double inversion, water suppression, OVS and FSI pulses. The FSI chemical shift (invppm) is user selectable (for Glu, invppm is 2.35 ppm) and the sequence automatically calculates the offset frequencies for the control and Selinv acquisitions to be symmetrically placed around water resonance (at 4.7 + 2.35 ppm) and at 2.35 ppm respectively. Inversion at (4.7 + 2.35 ppm) is done to remove any residual baseline water difference. For the SVS PRESS block, custom designed Shinnar-Le Roux (SLR) RF pulses, were used for uniform excitation and refocusing profile [19]. Relevant SLR pulse design parameters are: (a) excitation pulse: pulse width = 2.7 ms, time bandwidth product = 9, pass band ripple = 1 %, stop band ripple = 0.2 %, flip angle = 90°, and (b) refocusing pulse: pulse width = 6 ms, time bandwidth product = 12, pass band ripple = 1 %, stop band ripple = 0.1 %, flip angle = 150°. In the PRESS part of the sequence, for the water acquisition the excitation and refocusing pulses were set on resonance with water peak at 4.7 ppm while for all “water-suppressed” and “water and metabolite” spectra, the excitation and refocusing pulses were set at the glutamate chemical shift of 2.35 ppm to avoid chemical shift artifact for glutamate. This chemical shift based offset was added to the gradient strength and position-based frequency offset for all PRESS pulses. This ensures that the signal for water reference, Control1, Control2, Selinv1 and Selinv2 spectra all originate from the same location. The scan protocol uses a smaller number of averages for the water reference acquisition block and a larger number of averages for the water-suppressed and water-suppressed metabolite nulled acquisition blocks to optimize scan time.

### MR scan protocol

All the single voxel ^1^H MRS experiments were performed on a 7.0T whole body MRI scanner (Siemens Medical Systems, Erlangen, Germany) with a vendor supplied volume coil transmit/32-Channel receive proton head phased array coil. The acquisition protocol consisted of a gradient echo localizer, a T_1_-weighted three-dimensional (3D) magnetization prepared rapid gradient echo (MPRAGE) of whole brain. Localized automated first order and second order shimming of the B_0_ field was performed on the voxel of interest in order to obtain a water line width of <15 Hz (0.05 ppm) or less using FASTMAP shim method [20, 21] provided by Siemens as work in progress (WIP) package. Optimal reference voltage for the region of interest was calculated by acquiring two water spectra with reference voltages of 100 and 200 V using stimulated echo acquisition mode (STEAM) sequence. Assuming the peak amplitudes of water signal to be S1 and S2 for the above two reference voltages, the optimal reference voltage “B1” for the region of interest was calculated using the following equation:

$$B1 = \frac{90*100}{{\frac{180}{\pi }*{\text{acos}}\left(\sqrt[3]{{\frac{S2}{8*S1}}}\right) }}$$

Single voxel ^1^H MRS was obtained with FAMOUS SVGS sequence.

### Phantom studies

We verified the accuracy of the FAMOUS SVGS method using the glutamate monosodium phantoms. A stock solution of 100 mM Glutamate monosodium phantom was prepared using PBS buffer. From this stock solution, four phantoms were prepared at physiological pH to give final concentrations of 6, 9, 12, 15 mM. Even though with phantoms we could optimize shims to get line widths <5 Hz, to mimic the in vivo conditions, shim currents were mis-set to obtain a line width comparable to that of in vivo (15 Hz). Single voxel ^1^H MR spectra were obtained on the phantom with FAMOUS SVGS at room temperature using following parameters: voxel-size = 20 × 30 × 20 mm^3^, repetition time (TR) = 3000 ms and time to echo (TE) = 21 ms, number of points = 4096, sweep width = 6000 Hz, dummy scans = 4, averages = 8 (water)/32 (Control 1)/32 (Selinv1). Total acquisition time to obtain the spectra was 4 min:12 s per phantom.

### Human studies

#### Ethics, consent and permission

All the human studies were conducted under an approved University of Pennsylvania Institutional Review Board protocol with written informed consent obtained from each volunteer after explaining the study protocol.

Voxel was positioned in the occipital cortex (OCC, 20 × 30 × 20 mm^3^) with saturation band covering the skull area and the spectra was obtained with the following parameters: repetition time (TR) = 3000 ms and time to echo (TE) = 21 ms, number of points = 4096, sweep width = 6000 Hz, dummy scans = 4, averages = 8 (water), 32 (Control 1), 32 (Selinv1), 32 (Control 2), 32 (Selinv2). Total acquisition time to obtain the spectra was 7 min:48 s. All the five volunteers (all males, aged 19–62 years) were scanned twice within day for repeatability studies.

### Voxel placement for studies involving multiple scans

We used our in-house developed software, “Imscribe”, described elsewhere [22] to maintain the consistency in voxel placement for multiple acquisitions of ^1^H MRS from the same voxel.

**Fig. 2:**
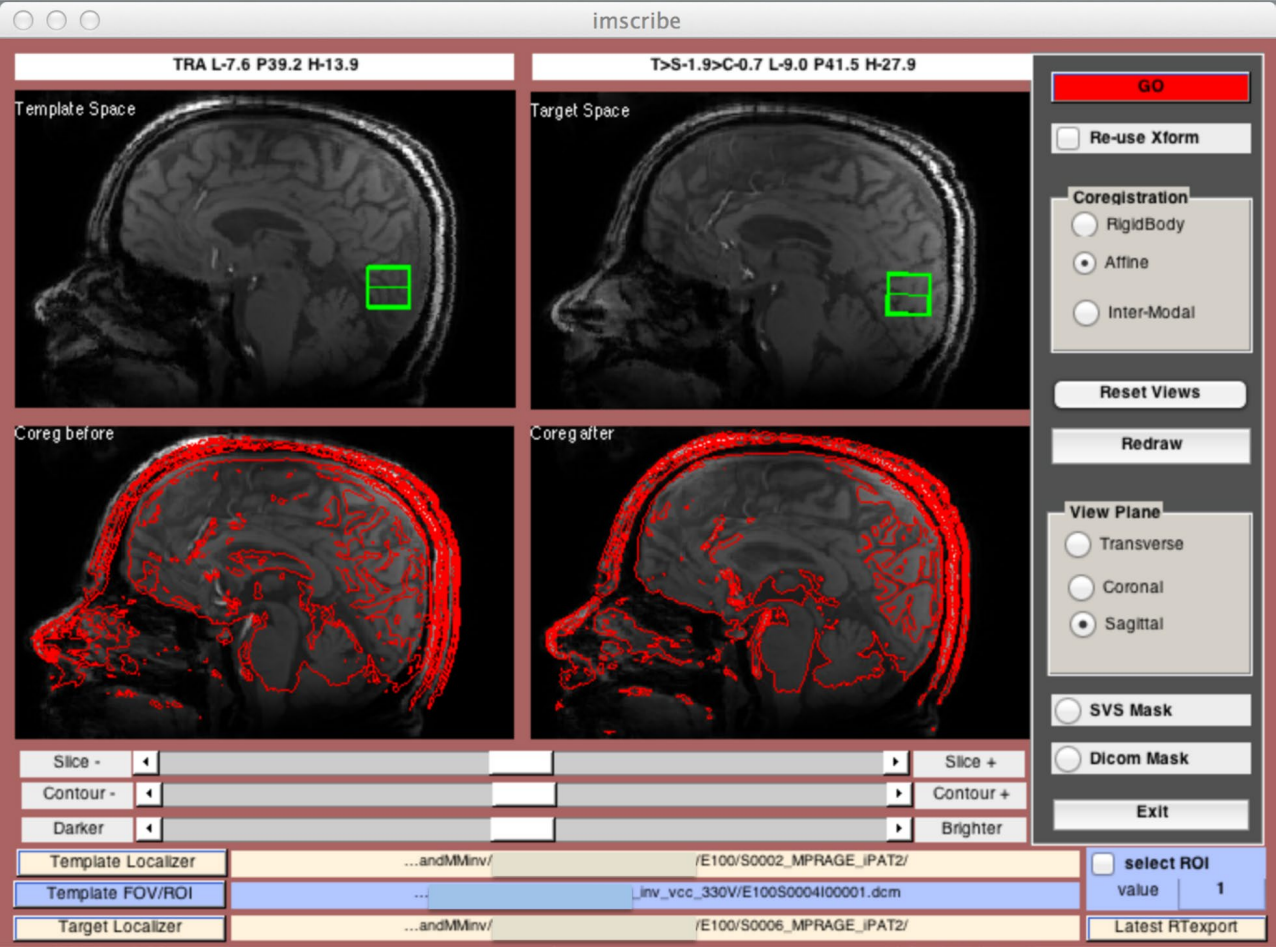
Snap-shot of the “Imscribe” software showing the co-registration process of the template and target T_1_-weighted images along with the new voxel information in the target space

Imscribe (accessible at: cmroi.med.upenn.edu/imscribe), uses high resolution T_1_-weighted images and the voxel information from the spectroscopy file acquired from first scan as target template for subsequent scans and performs affine co-registration giving the information for the new voxel placement as illustrated in Fig. 2.

### MRS data processing

**Fig. 3:**
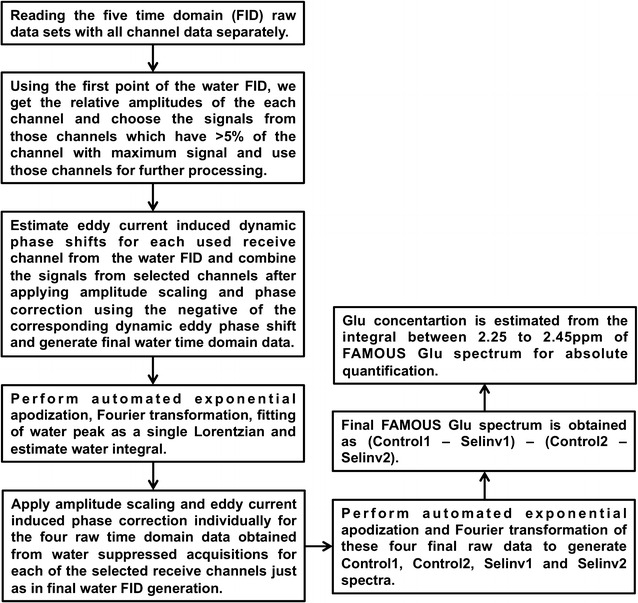
Flow chart showing the automated post-processing steps involved in quantification of the raw data obtained from the application of FAMOUS SVGS

For spectroscopy post-processing, we have an in-house developed automated post-processing package using MATLAB (R2014a, MathWorks, Natick, MA). Post-processing was done on the raw data from the system before any phased array channel combination and the flow chart is depicted in Fig. 3. For the phantom spectra, the FAMOUS Glu spectrum was obtained as (Control 1-Selinv1) and fitted as Lorentzian. Water content was assumed to be 100 % for phantoms.

In-house developed spectral processing method for FAMOUS SVGS works in the frequency domain. In this method, an exponential apodization of ~7 Hz was applied to the final time domain data after taking care of eddy current induced phase shifts, followed by automated Fourier transformation, phase correction and macromolecule removal. For fitting of water reference spectrum, we used non-linear least squares sense fitting (MATLAB “lsqcurvefit” routine). The integral of the Glu peak from 2.25 to 2.45 ppm (based on the inversion pulse bandwidth of 60 Hz) was normalized by water reference integral for absolute quantification of Glu. Water content was assumed to be 80 % for human brains.

### Statistics

Repeatability intra-class correlation coefficient (ICC) with 95 % confidence intervals (CI) values of single measures for the test–retest data was calculated with one-way random model using the SPSS software [version 18]. ICC value of <0.0 is interpreted as poor agreement, 0.0–0.2 indicates slight agreement, 0.21–0.4 indicates fair agreement, 0.41–0.6 indicates moderate agreement, 0.61–0.8 indicates substantial agreement, whereas ICC >0.81 indicates an almost perfect agreement [23]. ICC with p value less than 0.05 is considered as statistically significant.

## Results

**Fig. 4:**
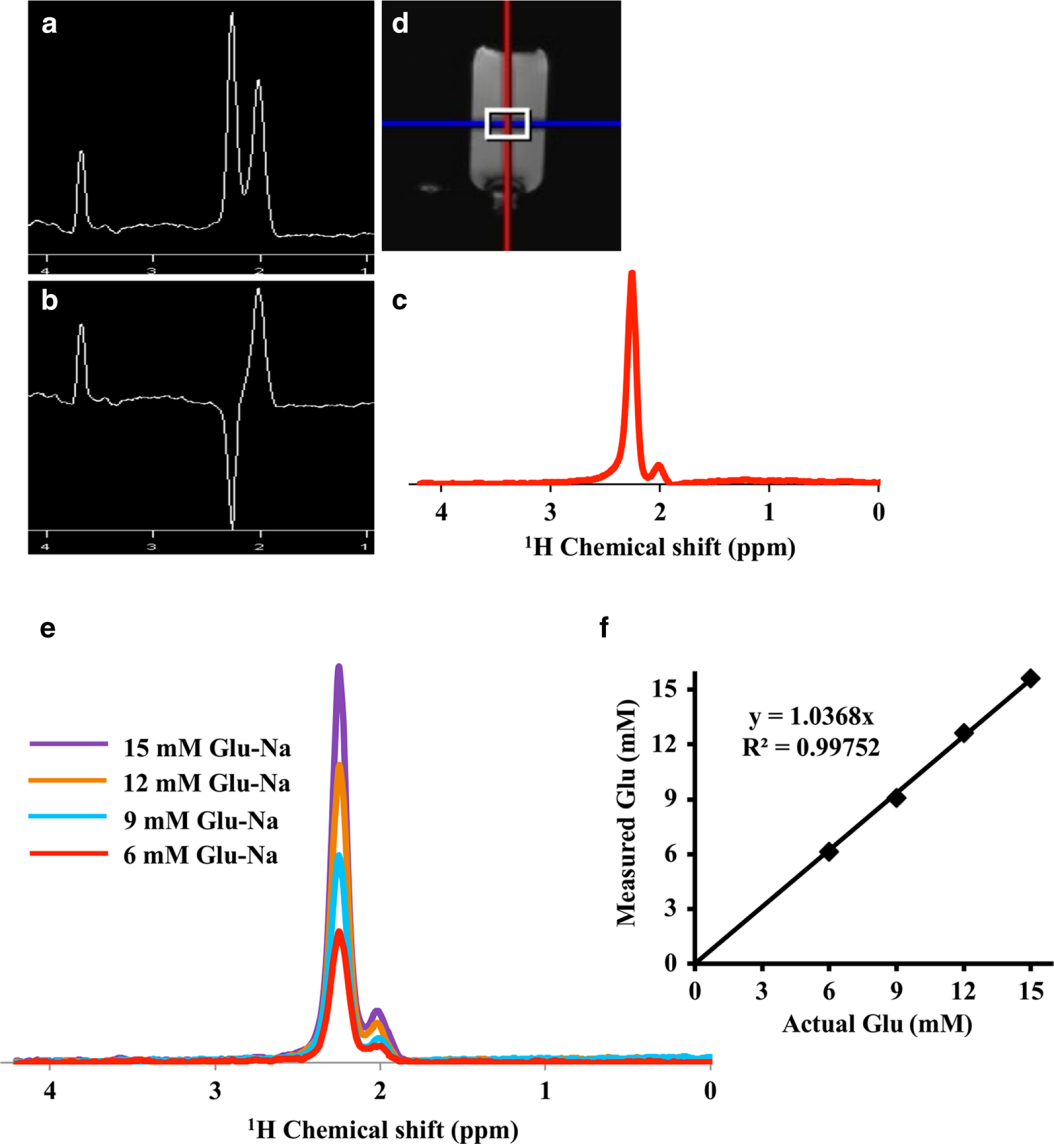
Water-suppressed control spectrum **(a)** and water-suppressed inversion spectrum **(b)** along with the FAMOUS Glu spectrum **(c)** obtained on region of interest shown on glutamate monosodium phantom image **(d)** along with the FAMOUS Glu spectra obtained from different concentrations of glutamate monosodium phantom **(e)** and a plot of actual vs measured Glu concentration **(f)** from FAMOUS Glu spectra

The concentration of glutamate from the voxel positioned on the glutamate monosodium phantom, as shown in Fig. 4, from the FAMOUS SVGS shows a good linear correlation between the actual and measured glutamate concentrations (slope = 1.03, goodness of fit as measured by R^2^ = 0.997).

**Fig. 5:**
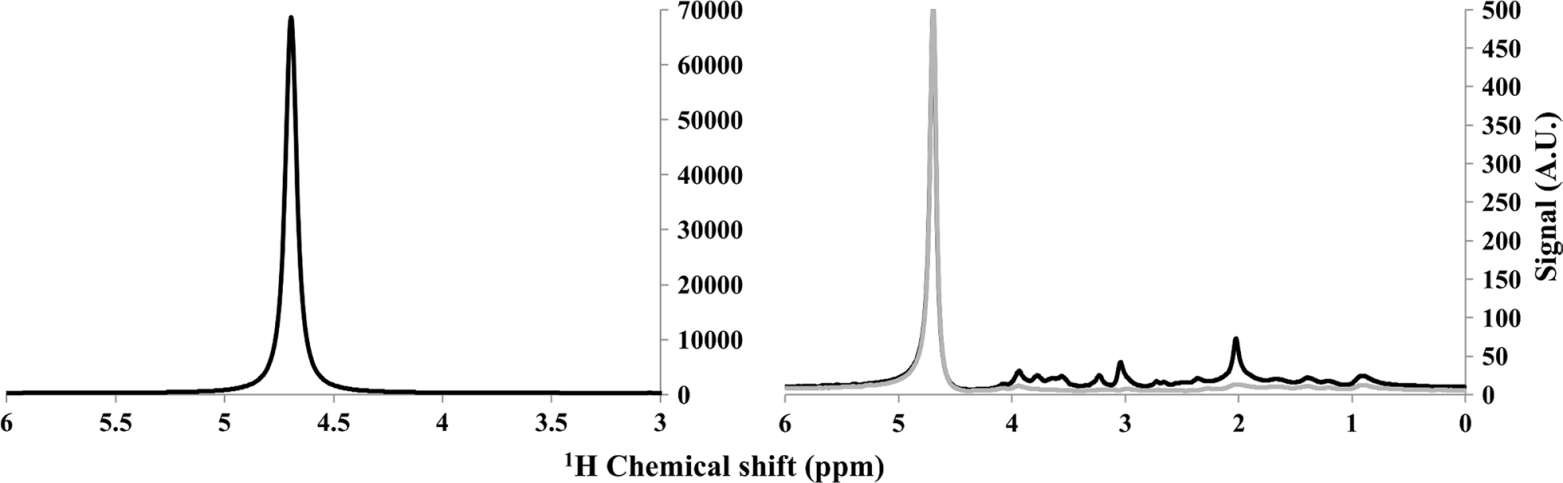
Typical water reference spectrum from one of the volunteer on OCC voxel along with the Control1 (*black*) and Control2 (*grey*) spectra demonstrating a good water suppression in the later two spectra. This suppression of water peak was 140-fold

Figure 5 shows the typical water spectrum obtained from the volunteer in OCC voxel and the typical Control1 and Control2 spectra. One can clearly see a 140-fold reduction in water signal in the later two spectra and metabolite suppression has very little effect on the quality of water suppression.

**Fig. 6:**
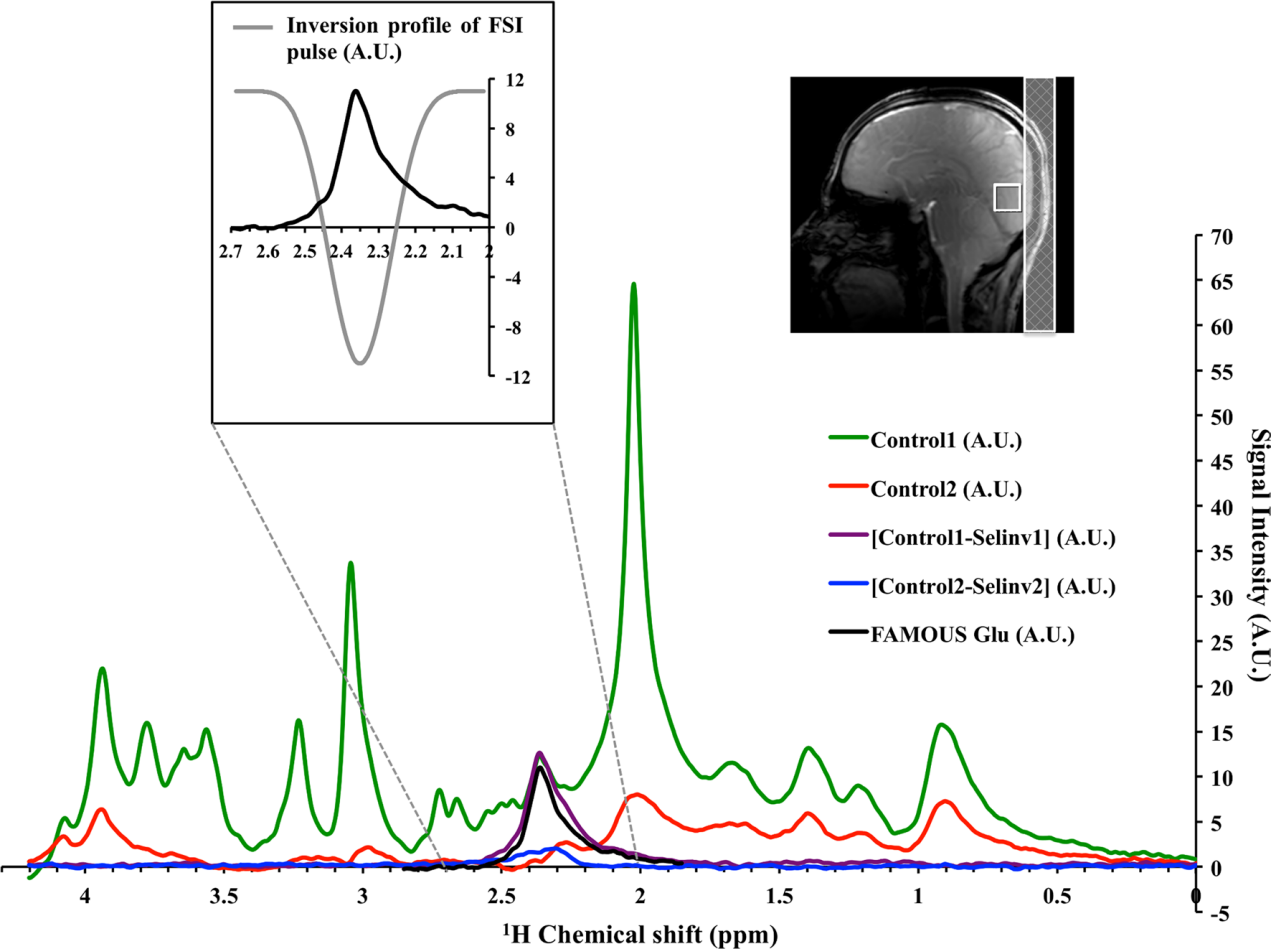
Anatomical position of the voxel on OCC from a volunteer is shown in T_1_-weighted image along with Control1 (*green*), Control2 (*red*), [Control1–Selinv1] (brown), [Control2-Selinv2] (*blue*) and FAMOUS Glu (*black*) spectra. In the inset is shown FAMOUS Glu spectrum along with the inversion profile (*grey*) of the FSI pulse at 2.35 ppm

Figure 6 shows the OCC voxel position and the typical Control1, Control2, (Control1-Selinv1), (Control2-Selinv2) and FAMOUS Glu spectra from a volunteer. In the inset is shown the FAMOUS Glu spectrum along with the inversion profile of the FSI pulse. FAMOUS Glu spectrum is integrated from 2.25 to 2.45 ppm, which is based on the inversion bandwidth of the FSI pulse as shown in inset of Fig. 6.

**Fig. 7:**
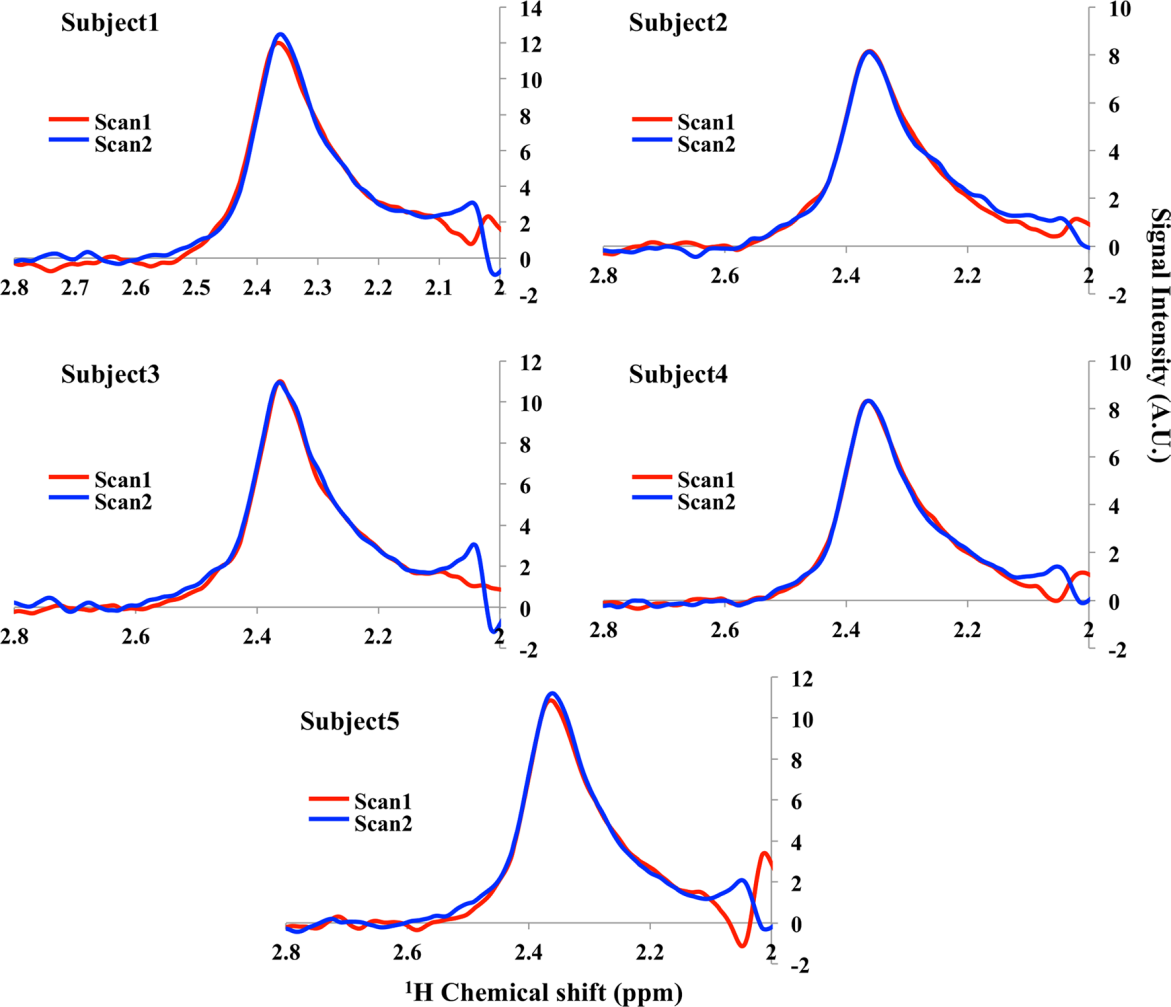
FAMOUS Glu spectra of scan1 (*red*) and scan2 (*blue*) of each volunteer are shown for all the five volunteers participated in the study

FAMOUS Glu spectra of test–retest of all the five subjects for each scan are shown in Fig. 7. The mean and standard deviation of Glu concentration from FAMOUS Glu spectra for the ten scans obtained from the five volunteers in the OCC voxel is 9.57 ± 0.63 mM; [CoV = 6.6 % calculated as (standard deviation/mean) × 100].

Absolute changes in Glu variations for each volunteer on OCC voxel are summarized in Table 1. The maximal absolute change in glutamate estimation observed from this voxel is <0.3 mM (ICC 0.961; 95 % CI LB 0.745, UB 0.996; p < 0.0005).

**Table 1:** Absolute variations in Glu concentrations for all the scans from FAMOUS Glu spectra, analyzed with in-house developed software

Subject no.	Glu (mM)		Abs Glu variation b/n scans
	Scan 1	Scan 2	
1	8.99	8.92	0.07
2	9.35	9.2	0.15
3	8.93	9.18	0.25
4	10.44	10.28	0.16
5	10.08	10.32	0.24

## Discussion

We have successfully demonstrated the feasibility of FAMOUS SVGS with studies on glutamate phantoms at 7.0T. The major advantage of this method is the fully automated processing with no user intervention for either phasing or baseline correction. We have also shown that we can estimate Glu concentrations in vivo from the data obtained from healthy subjects by FAMOUS SVGS with an intra-subject variability of less than 0.3 mM. This is most likely due to the simplification of the spectrum with negligible baseline and reduction of lipid/macromolecular contamination. We do find that the Glu concentrations in the occipital cortex from the FAMOUS SVGS are comparable to the previously published results from human brain autopsy study [24].

One of our goals in the current study was to bring down the scan-to-scan variation of Glu measurements to less than 0.5 mM. This objective was achieved with the FAMOUS SVGS method for our voxel of interest.

Although the FAMOUS SVGS method seems to be simplistic with the incorporation of a frequency selective inversion pulse, one might raise the following concern: (i) Effect of T_1_ recovery of Glu due to delay between inversion pulse and PRESS sequence: The reported T_1_ values of the metabolites at 7.0T were on the order of 1.21–1.83 s [25]. Therefore, the T_1_-weighting of the MR signals from the 12 ms delay (half the inversion pulse width = 10 ms and gradient crusher = 2 ms) between the inversion pulse and the PRESS sequence is negligible (<1 %) (ii) Macromolecule/lipid contamination at 2.35 ppm: This has been accounted in our method.

For the voxels of interest where local B_0_ inhomogeneity is large, we expect the accuracy of quantification to deteriorate due to poor shimming. Additionally, this method is highly sensitive to subject motion and hence we needed to immobilize the head with proper padding.

An alternate strategy we have considered was the use of long echo time without any inversion to reduce the macromolecule/lipid content from the control in vivo spectra. But the complicated coupling pattern of the glutamate peak poses additional quantification issues due to which we preferred to go along with the short echo spectroscopy with FAMOUS SVGS.

Although we have demonstrated the performance of FAMOUS SVGS in combination with in-house developed automated post-processing method at 7.0T, it can be easily implemented on animal imaging scanners ≥7.0T.

## Conclusions

In conclusion, FAMOUS SVGS can be one of the useful ^1^H MRS tools in any studies that measure regional glutamate changes in brain. The significant improvement in repeatability of the measurement has implications in quantifying relatively smaller change in Glu, reducing the number of subjects and time points needed in a clinical trial and thereby contributing to significant savings in study cost and time.

## Abbreviations

^1^H MRS: proton magnetic resonance spectroscopy
Glu: glutamate
SVGS: single voxel glutamate spectroscopy
SNR: signal-to-noise ratio
GABA: gamma-amino butyric acid
OVS: outer volume suppression
FSI: frequency selective inversion
PRESS: point resolved spectroscopy
RF: radio frequency
OCC: occipital cortex

## References

[1] Sutoo D, Akiyama K, Yabe K (2000). Quantitative maps of GABAergic and glutamatergic neuronal systems in the human brain. Hum Brain Mapp.

[2] Auer DP, Pütz B, Kraft E, Lipinski B, Schill J, Holsboer (2000). Reduced glutamate in the anterior cingulate cortex in depression: an in vivo proton magnetic resonance spectroscopy study. Biol Psychiatry.

[3] Benedetti F, Calabrese G, Bernasconi A, Cadioli M, Colombo C, Dallaspezia S, Falini A, Radaelli D, Scotti G, Smeraldi E (2009). Spectroscopic correlates of antidepressant response to sleep deprivation and light therapy: a 3.0 Tesla study of bipolar depression. Psychiatry Res.

[4] Griffith HR, den Hollander JA, Okonkwo OC, O’Brien T, Watts RL, Marson DC (2008). Brain metabolism differs in Alzheimer’s disease and Parkinson′s disease dementia. Alzheimers Dement.

[5] Gutzeit A, Froehlich JM, Hergan K (2012). Insula-specific ^1^H magnetic resonance spectroscopy reactions in heavy smokers under acute nicotine withdrawal and after oral nicotine substitution. Eur Addict Res.

[6] Hasler G, van der Veen JW, Tumonis T, Meyers N, Shen J, Drevets WC (2007). Reduced prefrontal glutamate/glutamine and gamma-aminobutyric acid levels in major depression determined using proton magnetic resonance spectroscopy. Arch Gen Psychiatry.

[7] Kakeda S, Korogi Y, Moriya J, Ohnari N, Sato T, Ueno S, Yanagihara N, Harada M, Matsuda T (2011). Influence of work shift on glutamic acid and gamma-aminobutyric acid (GABA): evaluation with proton magnetic resonance spectroscopy at 3T. Psychiatry Res.

[8] Kegeles LS, Mao X, Stanford AD (2012). Elevated prefrontal cortex γ-aminobutyric acid and glutamate-glutamine levels in schizophrenia measured in vivo with proton magnetic resonance spectroscopy. Arch Gen Psych.

[9] Marsman A, van den Heuvel MP, Klomp DW, Kahn RS, Luijten PR, Pol HHE (2013). Glutamate in schizophrenia: a focused review and meta-analysis of ^1^H-MRS studies. Schizophr Bull.

[10] Petroff OA, Rothman DL, Behar KL, Mattson RH (1995). Initial observations on effect of vigabatrin on in vivo ^1^H spectroscopic measurements of gamma-aminobutyric acid, glutamate, and glutamine in human brain. Epilepsia.

[11] Price RB, Shungu DC, Mao X, Nestadt P, Kelly C, Collins KA, Murrough JW, Charney DS, Mathew SJ (2009). Amino acid neurotransmitters assessed by proton magnetic resonance spectroscopy: relationship to treatment resistance in major depressive disorder. Biol Psychiatry.

[12] Sanacora G, Gueorguieva R, Epperson CN, Wu YT, Appel M, Rothman DL, Krystal JH, Mason GF (2004). Subtype-specific alterations of gamma-aminobutyric acid and glutamate in patients with major depression. Arch Gen Psychiatry.

[13] Schmaal L, Veltman DJ, Nederveen A, van den Brink W, Goudriaan AE (2012). N-acetylcysteine normalizes glutamate levels in cocaine-dependent patients: a randomized crossover magnetic resonance spectroscopy study. Neuropsychopharmacology.

[14] Yucel M, Lubman DI, Harrison BJ (2007). A combined spectroscopic and functional MRI investigation of the dorsal anterior cingulated region in opiate addiction. Mol Psychiatry.

[15] Stephenson MC, Gunner F, Napolitano A, Greenhaff PL, Macdonald IA, Saeed N, Vennart W, Francis ST, Morris PG (2011). Applications of multi-nuclear magnetic resonance spectroscopy at 7T. World J Radiol.

[16] Cai K, Nanga RPR, Lamprou L, Schinstine C, Elliott M, Hariharan H, Reddy R, Epperson CN (2012). The impact of gabapentin administration on brain GABA and glutamate concentrations: a 7T ^1^H-MRS study. Neuropsychopharmacology.

[17] Terpstra M, Cheong I, Lyu T, Deelchand DK, Emor UE, Bednarik P, Eberly LE, Oz G (2015). Test-retest reproducibility of neurochemical profiles with short-echo, single-voxel MR spectroscopy at 3T and 7T. Magn Reson Med.

[18] Lally N, An L, Banerjee D, Niciu MJ, Luckenbaugh DA, Richards EM, Roiser JP, Shen J, Zarate CA, Nugent AC (2016). Reliability of 7T (1)H-MRS measured human prefrontal cortex glutamate, glutamine, and glutathione signals using an adapted echo time optimized PRESS sequence: a between- and within-sessions investigation. J Magn Reson Imaging.

[19] Pauly J, LeRoux P, Nishimura D, Macovski A (1991). Parameter relations for the Shinnar-Le Roux selective excitation pulse design algorithm [NMR imaging]. IEEE Trans Med Imaging.

[20] Gruetter R (1993). Automatic, localized in vivo adjustment of all first—and second-order shim coils. Magn Reson Med.

[21] Gruetter R, Tkac I (2000). Field mapping without reference scan using asymmetric echo-planar techniques. Magn Reson Med.

[22] Wolf DH, Satterhwaite TD, Loughead J, Pinkham A, Overton E, Elliott MA, Dent GW, Smith MA, Gur RC, Gur RE (2011). Amygdala abnormalities in first-degree relatives of individuals with schizophrenia unmasked by benzodiazepine challenge. Psychopharmacology.

[23] Landis JR, Koch GG (1977). The measurement of observer agreement for categorical data. Biometrics.

[24] Perry TL, Berry K, Hansen S, Diamond S, Mok C (1971). Regional distribution of amino acids in human brain obtained at autopsy. J Neurochem.

[25] Xin L, Schaller B, Mlynarik V, Lu H, Gruetter R (2013). Proton T1 relaxation times of metabolites in human occipital white and gray matter at 7T. Magn Reson Med.

